# A Multifaceted Intervention to Reduce Obesity Among Preschool Children in Suzhou City: Protocol for a Randomized Controlled Trial

**DOI:** 10.2196/94697

**Published:** 2026-10-21

**Authors:** Pan Yang, Ying Wang, Xiao Jiang, Ziyao Ding, Liye Zhang, Xinyu Bao, Di Han, Hui Shen, Jia Hu, Jieyun Yin

**Affiliations:** 1 Department of Epidemiology and Health statistics, Suzhou Medical School, Soochow University, Suzhou, Jiangsu, China; 2 Department of Child and Adolescent Health Promotion, Suzhou Center for Disease Control and Prevention (Suzhou Health Supervision Institute), 16 Guangqian Road, Suzhou, Jiangsu, 215131, China, 86 512-62701715; 3 Suzhou Institute of Advanced Study in Public Health, Gusu School, Nanjing Medical University, Suzhou, Jiangsu, China; 4 School of Public Health, Nanjing Medical University, Nanjing, Jiangsu, China

**Keywords:** children, obesity, multifaceted intervention, parents, community health care, school, family health care

## Abstract

**Background:**

The rising prevalence of obesity among preschool children has become a critical public health concern in Suzhou, China. Nevertheless, evidence-based, personalized, and multicomponent intervention strategies tailored to children with overweight or obesity remain inadequately established in current practice.

**Objective:**

This study aims to investigate the prevalence and pathogenic factors of obesity among preschool children in Suzhou and to develop a comprehensive and multifaceted integrated intervention program. Through systematic assessment of its short-term effectiveness and long-term sustainability in improving obesity-related indicators and preventing overweight, it provides a scientific basis for obesity prevention and control.

**Methods:**

This cluster randomized controlled trial is being conducted among children aged 3 to 6 years in Suzhou City, Jiangsu Province. Children from 3 kindergartens were randomly assigned to the intervention group and children from 3 other kindergartens were assigned to the control group. The 1-year intervention includes health education, dietary guidance, and increased physical activity, adopting a multilevel approach that engages children, families, schools, communities, and society. Children participate in health education and physical activity courses in the kindergarten, while parents receive educational materials online. Parental education and regular assessment of knowledge, attitudes, and practices are key components of the family-based intervention. Body measurements will be taken at baseline, 6 months, 12 months, and thereafter, alongside questionnaires for both parents and children to evaluate their own health behaviors. The primary outcome will be the difference in BMI, BMI *z* scores, and body fat percentage between the 2 groups of children at the end of the intervention.

**Results:**

The study received ethical approval in March 2025. Baseline assessments were conducted from March to June 2025, followed by the initiation of the intervention, which will be completed by June 2026. Participants will then enter a noninterventional long-term follow-up until June 2032, with annual surveys conducted during this period.

**Conclusions:**

This program will test the effectiveness of comprehensive multifaceted interventions on children. The results of this study will provide scientific evidence for formulating effective intervention strategies for childhood overweight and obesity and are also expected to offer crucial practical references for the formulation and improvement of treatment guidelines for childhood obesity in China.

## Introduction

The increasing prevalence of childhood overweight and obesity has emerged as a critical public health challenge [1]. Globally, the proportion of children with overweight aged 2 to 4 years rose from 15.50% in 2012 to 19.11% in 2021, with projections reaching 22.32% by 2030 [2]. China, in particular, has witnessed one of the most rapid increases in childhood overweight and obesity, especially among preschool-aged children [3]. Overweight and obesity, defined as an abnormal or excessive accumulation of fat, are considered complex, chronic, and prone to recurrence and are now recognized as persistent conditions that can extend from childhood into adulthood [4,5]. Affected children face heightened risks for dyslipidemia, hypertension, diabetes mellitus, nonalcoholic fatty liver disease, obstructive sleep apnea, psychosocial disturbances, impaired quality of life, and shortened life expectancy [6-9]. Early intervention is therefore essential to prevent further increases in childhood obesity rates and related health hazards [9,10].

In Suzhou, the obesity rate among preschool children is 14%, exceeding the 10.4% reported in the *Report on Nutrition and Chronic Diseases of Chinese Residents* (2020) [11]. The preschool stage (ages 3-6 years) is a critical period for the formation and consolidation of lifestyle habits such as a healthy diet and physical activity, which have a certain degree of persistence and are closely related to the risk of childhood overweight and obesity [12,13]. Therefore, targeted interventions during this formative period are vital for effective obesity prevention in Suzhou.

While previous studies have suggested interventions such as dietary adjustments and exercise promotion [14,15], these efforts are often fragmented and lack systematic integration, which limits their effectiveness in tackling the complex causes of childhood obesity. Furthermore, even comprehensive intervention studies have notable gaps. First, parent-related outcomes, particularly the changes in parents’ knowledge, attitudes, and behaviors regarding childhood obesity and healthy lifestyles, have been reported relatively infrequently in previous studies [16,17]. Many parents continue to view childhood obesity as a temporary condition that will resolve with age [18], underestimating its lasting dangers. Meanwhile, the family environment is crucial for sustaining healthy behaviors. Therefore, it is essential to evaluate and support parents’ understanding, attitudes, and behaviors to foster a positive setting for children.

Second, the follow-up evaluations of existing intervention studies for preschool children with obesity mainly focus on weight-related indicators such as BMI or BMI *z* score and rarely include body composition indicators such as body fat percentage (BFP), soft lean mass, skeletal muscle mass, and total, trunk, arm, and leg fat-free mass [19-21]. Therefore, they may not fully reflect the specific changes in fat tissue and lean tissue after the intervention. This limited focus may miss children with normal BMI but unhealthy fat distribution, resulting in an incomplete evaluation of intervention effectiveness and potentially underestimating long-term management needs. Finally, most current studies lack extended follow-up, with intervention and assessment periods typically lasting only 6 months [22], which limits understanding of the durability of intervention effects over time.

The Social Ecological Model (SEM) offers a comprehensive framework for understanding the multiple levels of influence on childhood obesity. Children’s weight status results from a complex interaction of personal factors (such as diet and physical activity), interpersonal influences (including family eating habits and peer behaviors), institutional settings (like school food policies and physical education), and broader community and policy factors (such as neighborhood walkability and food availability) [23-26]. Multicomponent and multilevel ecological intervention strategies, integrating children’s health education with physical activities, family participation, and environmental support in kindergartens or schools, are therefore considered effective [27,28]. Therefore, guided by SEM, we designed intervention measures that address these diverse influences to effectively reduce childhood obesity and promote overall health.

Complementing the SEM, Social Cognitive Theory (SCT) focuses on the cognitive and motivational processes that drive behavioral change, with particular emphasis on cultivating self-efficacy. Unlike other theories of behavior change, SCT places greater importance on maintaining positive behavior through self-regulation and reinforcement, rather than merely initiating change [29]. Previous interventions for childhood obesity have utilized SCT to establish a behavioral change path centered on enhancing the self-efficacy and self-regulation abilities of both children and their parents [30,31]. In our intervention, we incorporated SCT-based strategies such as goal setting, self-monitoring, and problem-solving skills training, while reinforcing healthy behaviors through role models and social support from peers, families, and teachers [32-34].

By integrating SEM and SCT, our approach provides a comprehensive, multilevel strategy that addresses both environmental and psychological factors to reduce childhood obesity and support lasting health improvements in Suzhou. The primary objectives of this study are (1) to assess the prevalence of obesity among preschool children in Suzhou and analyze the main risk factors; (2) to reduce the proportion of children with obesity through comprehensive interventions and evaluate the effectiveness of these interventions compared to routine care; and (3) to determine the sustainability of the intervention in preventing overweight and obesity.

## Methods

### Design

This study employs a cluster randomized controlled trial design across 6 kindergartens in Suzhou, one of the most socioeconomically developed cities in China. Schools are randomly assigned to either the intervention or control group, with randomization conducted postbaseline assessment to ensure allocation concealment. The study flowchart is displayed in Figure 1, and Table 1 summarizes the study process. A completed SPIRIT 2025 checklist is provided as Checklist 1, detailing the corresponding page references within this protocol.

**Figure 1.**
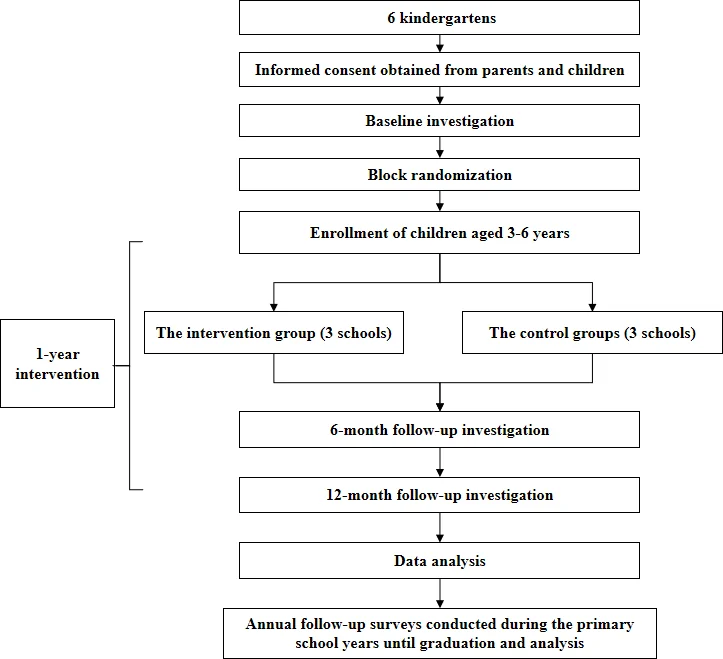
Flowchart of participants in the trial.

**Table 1.** Participant timeline according to SPIRIT^a^.

	Trial period									
	Enrollment	Baseline	Postrandomization		Postintervention					
Time point	*−t_i_* to 0	0	*t*_1_ ^b^	*t*_2_ ^c^	*t*_3_ ^d^	*t*_4_ ^e^	*t*_5_ ^f^	*t*_6_ ^g^	*t*_7_ ^h^	*t*_8_^i^ (close out)
Enrollment										
Eligibility screen	✓									
Informed consent		✓								
Randomization		✓								
Interventions										
Intervention		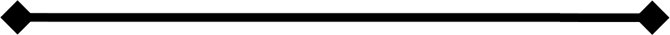								
Control		✓								
Assessments										
Anthropometric data		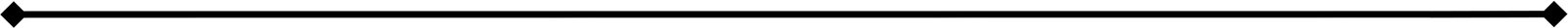								
Bioimpedance data		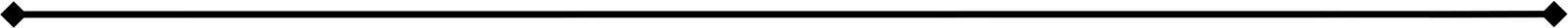								
Child well-being		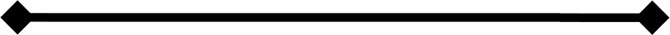					✓			✓
Child eating habits		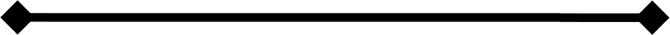					✓			✓
Child physical activity		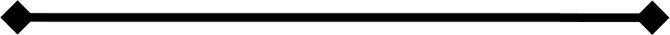					✓			✓
Child screen use		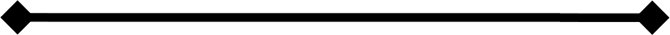					✓			✓
Child sleep		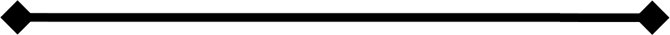					✓			✓
Family food and meals		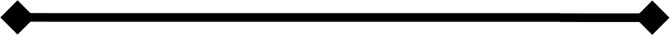					✓			✓
Family physical activity		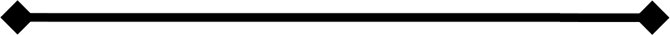					✓			✓
Family screen use		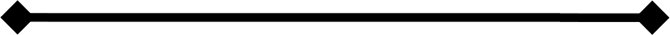					✓			✓
Healthy knowledge		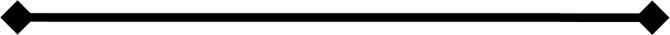					✓			✓

a SPIRIT: Standard Protocol Items: Recommendations for Interventional Trials.

b *t*_1_: 6 months after baseline.

c *t*_2_: 12 months after baseline.

d *t*_3_: 24 months after baseline.

e *t*_4_: 36 months after baseline.

f *t*_5_: 48 months after baseline.

g *t*_6_: 60 months after baseline.

h *t*_7_: 72 months after baseline.

i *t*_8_: 84 months after baseline.

This study comprises 2 distinct phases. The first phase is the main intervention study, which includes study preparation, a baseline survey, intervention implementation, and 12-month outcome evaluation. This phase mainly evaluates the short-term effects of the comprehensive intervention on preschool children’s weight, body composition, physical fitness, and related health behaviors. The second phase is a planned extension observation following the first phase. This will not continue the original kindergarten intervention but will investigate whether the effects observed during the preschool period can persist into school age. Long-term extension follow-up will complete the measurements stipulated in the main intervention study while adhering to the ethical requirements and informed consent procedures.

### Participants and Eligibility Criteria

At the beginning, the study plan was disclosed, and the Centers for Disease Control and Prevention in each district were consulted for input on the research proposal. Then, the Centers for Disease Control and Prevention authorities provided essential information about potential participating schools, including school type, number of children, and number of teachers. Next, the research team assessed each school’s circumstances and conducted on-site evaluations to finalize school selection. At last, the lead researcher compiled a list of eligible schools and classes, inviting these institutions to participate in the study. Since children in senior classes would graduate in the next midyear, but the intervention program is designed to last a full year, the project will focus on junior and middle classes in selected kindergartens.

The selection of participating schools was based on the following criteria: school administrators were interested in the program and agreed to implement it; the school had relevant staff (such as a school nurse or health practitioner, physical education teacher, or student cafeteria dietitian); the school was not a boarding school or a specialized school for minority children; and the school did not have, nor plan to implement, a similar intervention program. This study used cluster randomization at the kindergarten level, stratifying the 6 kindergartens by geographic location into a central urban stratum (2 kindergartens) and a surrounding districts stratum (4 kindergartens). Using a lottery method, 1 urban kindergarten and 2 from the surrounding areas were assigned to the intervention group, while the others formed the control group, matched based on size, economic status, and kindergarten attendance duration.

Children were eligible for inclusion if they were between 3 and 6 years old and their guardians had been informed and provided consent to participate in the study. Exclusion criteria included a history of heart disease, hypertension, diabetes mellitus, asthma, viral hepatitis, or nephritis; obesity due to endocrine disease or medication; abnormal physical development or deformities; inability to participate in school sports; or recent weight loss due to vomiting or medication use within the past 3 months.

### Screening and Recruitment Process

After the routine annual national physical examination at the beginning of the school year, all caregivers (primarily parents) of children in each school will be invited to attend a program orientation session. Written informed consent will be obtained from the primary caregivers of children who wish to participate. Physical measurements will then be conducted on children whose parents have provided consent (Figure 1).

### Intervention Procedures

Based on the SEM and SCT, this multicomponent, school-based, and family-involved program adopts a “community-school-family-medical” integrated prevention model. As shown in Figure 2, the program implements targeted interventions at individual, family, school, community, and societal levels to address childhood obesity and influence preschool children’s knowledge, attitudes, and behaviors.

**Figure 2.**
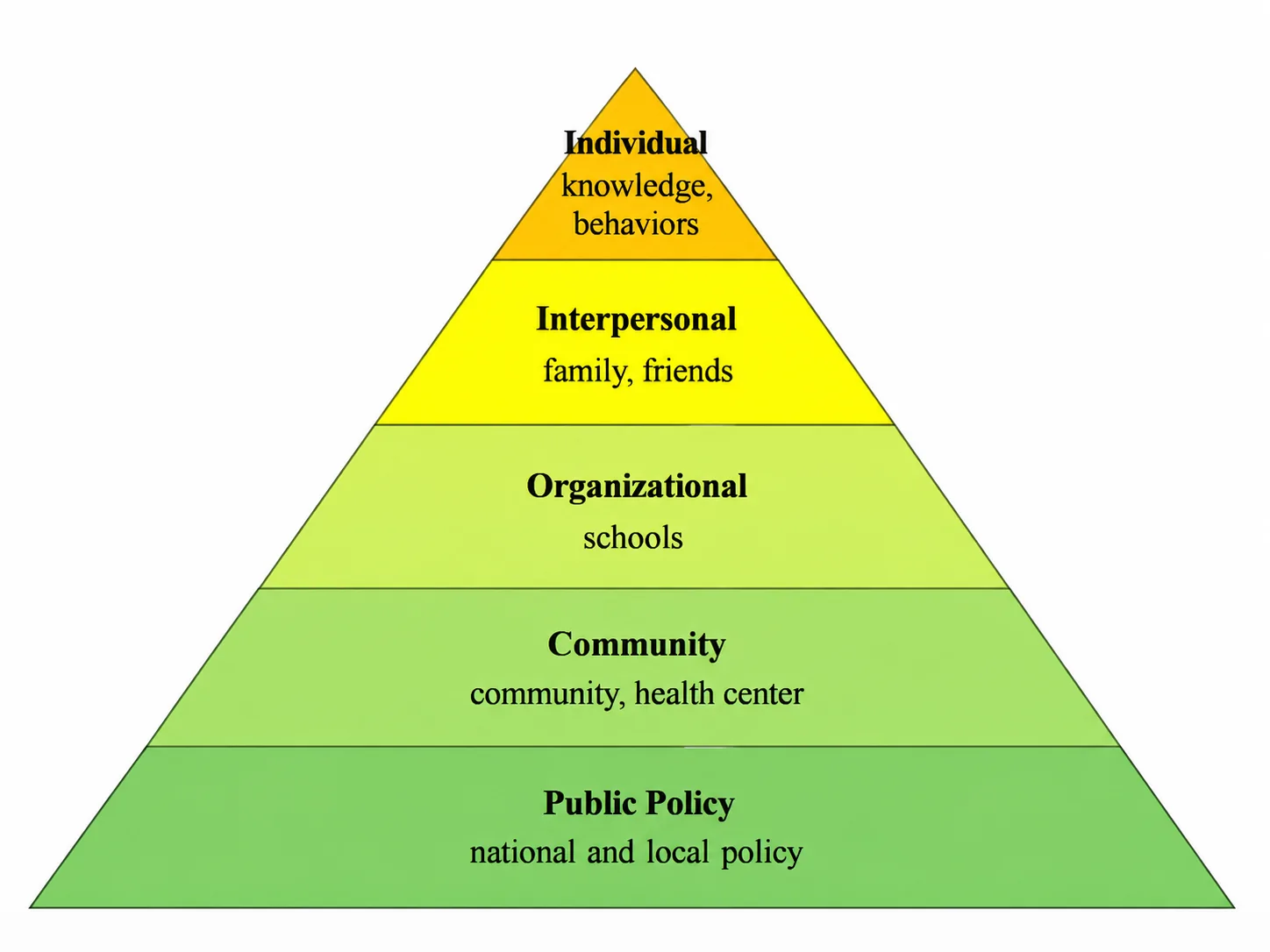
Framework of the intervention program.

Interventions will also be carried out during holidays. Trained professionals will distribute health materials to children and organize a 3-week check-in activity. During this period, children’s daily habits—including diet (regular meals, adequate water intake, no sugary beverages), exercise (outdoor activities, sedentary time, screen time), and sleep patterns—will be recorded. The aim is to help children gradually develop healthy habits.

Moreover, children with a tendency toward overweight or obesity and their parents will all join a “Move More” club. The club is composed of physical education teachers, health care doctors, head teachers, kindergarten principals, children with special needs, and their parents. The teachers in the club will provide additional guidance and assistance regarding weight management. The multicomponent intervention includes child-focused health education and specialized physical education delivered at the kindergarten, together with face-to-face and online educational activities for parents. Details of nonholiday intervention content, delivery mode, frequency, duration, and total dose are presented in Table 2.

**Table 2.** Specific intervention measures.

Intervention components	Descriptions of the content, frequency, and duration	Person responsible
1. Student-focused activities (health education activities for children)	Preschool children receive 10 health education sessions delivered by a trained teacher once a month throughout the intervention period, with each session lasting 20 minutes. The core content of the program is to recognize obesity and prevent it (including healthy diet, regular exercise, reducing snack intake, and getting sufficient sleep). Physical education teachers increase the amount or duration of exercise (mainly aerobic exercise) to more than 60 minutes in accordance with expert advice. Specialized physical sessions, each lasting 30 minutes, will be conducted 3 to 4 times per week over the 10-month intervention period to encourage children to engage in more physical activity. When the weather is inclement, teachers may guide children to do relevant alternative activities to maintain their physical activities, for example, ice skating in winter and indoor games on rainy days.	The trained teachers
2. Activities toward parents (providing a supportive family environment)	Parents should rationalize their children’s diet according to the “Dietary Guidelines on Childhood and Adolescent Obesity,” have regular meals every day, try not to eat in restaurants, reduce the consumption of sugary carbonated beverages, increase the intake of vegetables, and reduce the purchase of unhealthy snacks. Parents should take their children to do some appropriate outdoor activities every day according to the “Physical Education and Sports Guidebook,” and encourage them to engage in more games and communication with their peers. Limit children’s screen time, including not watching TV during meals. To change or maintain the parenting style and lifestyle of the family, to overcome traditional culture and the concept of overfeeding, to change the way of rewarding food and the behavior of overcontrolling food, and to maintain their own state of health or to improve the state of ill health, including physical and mental health. Parents of children with overweight or obesity all join a Move More club, and the teachers in the club will provide additional guidance and assistance regarding weight management.	Parents or custodians
3. Activities toward schools (providing a supportive school environment)	A professional dietitian assesses whether the kindergarten meal meets the national recommended requirements and will evaluate the meal pattern of each child based on a questionnaire, make recommendations, and help the cafeteria staff adjust the school lunch and afternoon tea once a month. Teachers need to measure children’s height and weight every month and give timely feedback to parents in addition to keeping records. Reward children with obesity or overweight whose BMI has dropped significantly by selecting little masters of weight management or little stars of weight control to encourage them. School administrators need to post posters and wall posters on weight management outside the classroom, not set up vending machines for sugary drinks outside the classroom, and provide water fountains to encourage drinking more water. Increase the number of sports facilities and playing fields, and organize fun sports activities every semester to attract children’s interest. Create a positive school climate and avoid stressors associated with a poor school climate such as bullying, personal safety, peer behavior, and weight stereotyping.	The trained class teachers and school administrators
4. Activities toward the community (providing a supportive community environment)	Face-to-face parent education seminars will be held at the kindergarten once per semester, with 2 sessions delivered in total. Each session should last approximately 1 hour and involve teaching specialists, physical education teachers, and parents. Professional knowledge and health manuals are provided, and after the seminars, the program professionals are added to the parents’ group to facilitate communication. Medical check-ups and assessments of children are conducted by professionals every 6 months, and medical assessments and guidance are provided. Posters of health knowledge are put up on community bulletin boards, the media will publicize healthy food, community stores will be encouraged not to provide unhealthy food to children, and the official WeChat public account will publish 2 high-quality scientific articles each month, accompanied by animated videos and case studies, in line with the slogan “Eat wisely, move wisely, prevent obesity.” The community needs to provide sports facilities for both adults and children to facilitate leisure sports for parents and children after work to enhance parent-child bonding, as well as for children to communicate and play with their buddies after completing homework.	Communities and professionals
5. Policy	Establish cross-sectoral collaboration, setting up a multidepartmental joint system of the Ministry of Education, the Health Planning Commission, and the General Administration of Market Supervision and Regulation, and jointly issuing the “Guidelines for Prevention and Control of Obesity in Preschool Children” to clarify the responsibilities of kindergartens, families, and communities. Incorporate “child-friendly exercise space” into neighborhood renovation indicators and require the addition of physical training facilities for young children. Establish mandatory health standards, require kindergartens to ensure preschool children’s activity levels and meals, and incorporate obesity prevention and control into kindergarten grade assessments.	Government

If any participant experiences a serious adverse event related to the study (eg, severe physical injury or psychological stress) during the intervention period, the intervention will be immediately stopped.

### Control Group

Schools in the control group will not implement any intervention components and will instead continue with their regular educational programs and physical exercises during the period according to local policies.

### Data Collection

During the study period, multiple data collection methods will be used to evaluate the effectiveness and impact of the intervention. These include (1) anthropometric measurements of children, (2) interviews with kindergarten teachers, and (3) parent questionnaires. Data will be collected from both the intervention and control groups. The following sections outline these measures and describe the data collection procedures in detail.

### Children’s Anthropometric Measurements

Comprehensive anthropometric measurements will be conducted at the start of the study, at 6 months, and at 12 months. Measurements will include height, weight, waist and hip circumference, body composition, systolic and diastolic blood pressure, and physical fitness indicators (grip strength, standing long jump, sitting forward flexion, continuous jump of both feet, 15-m obstacle run, and balance beam). Children’s physical fitness will be assessed according to the National Physical Fitness Testing Standard (revised in 2023) issued by the China National Physical Fitness Monitoring Center [35], which is applicable to children aged 3 to 6 years. Detailed testing procedures are presented in Table 3.

**Table 3.** Description of parameters and applied measurement methods.

Parameter	Method of measurement
Height	No shoes, headwear, or headwear accessories. Measure using an automatic height and weight gage with an accuracy of 0.5 cm.
Weight	Wear light clothing, no shoes, and no heavy objects in the pockets. Use the automatic height and weight measuring device for the measurement, with an accuracy of 0.1 kg.
Chest circumference	Chest circumference was measured with the child standing upright, arms at the sides, and feet shoulder-width apart. A tape was placed horizontally around the chest at the upper border of the nipples without compressing the skin. The measurement was recorded at the end of normal expiration to the nearest 0.1 cm, with an allowable error of ≤1 cm.
Waist circumference	Waist circumference was measured with the child standing naturally, arms relaxed, and feet 30‐40 cm apart. A tape was placed horizontally around the abdomen 1 cm above the umbilicus and at the midpoint between the lowest rib and the iliac crest. The measurement was taken during normal breathing, with an allowable error of ≤1.0 cm.
Hip circumference	Hip circumference was measured with the child standing naturally, shoulders relaxed, legs together, and arms at the sides. A tape was placed horizontally around the hips, passing over the pubic symphysis and the most prominent part of the buttocks.
Blood pressure	Blood pressure was measured using an electronic sphygmomanometer (Omron, Osaka, Japan) with the participant seated and the right arm supported at heart level. Two measurements were taken 1‐2 min apart, and the mean value was recorded. If the systolic or diastolic readings differed by more than 10 mm Hg, a third measurement was taken and the mean of all 3 readings was recorded.
Body composition	Body composition analysis was performed in a standing position. In light clothes, with no shoes, socks, tights, or heavy items in pockets. Measurement was taken using bioelectrical impedance analyzers (InBody 570). Before each measurement, the electrodes were thoroughly wiped with an appropriate disinfectant.
Grip strength	Grip strength was measured using a pediatric handgrip dynamometer (Xinman WCS-100 model, Shanghai, China). The child stood with feet shoulder-width apart and arms extended obliquely downward, then squeezed the dynamometer maximally with the dominant hand. Two measurements were taken and recorded to the nearest 0.1 kg.
Standing long jump	Standing long jump was assessed using a standardized test mat. The child jumped forward from behind the take-off line using both feet. Two trials were performed, and the distance was recorded in whole centimeters.
Sitting forward flexion	Sitting forward flexion was assessed using a standardized testing device. The child sat barefoot with the legs fully extended and feet against the footplate, then reached forward with both hands while keeping the knees straight. Two trials were performed, and the distance was recorded to the nearest 0.1 cm.
Continuous jump of both feet	Continuous jump of both feet was assessed using 10 soft blocks (10 × 5 × 5 cm) placed 50 cm apart along a 1.5 m course. The child jumped with both feet over all 10 blocks as quickly as possible. Two trials were performed, and completion time was recorded to the nearest 0.1 s.
15-m obstacle run	The 15-m obstacle run was assessed using 7 cones placed 1.5 m apart along the course. The child sprinted 3 m, weaved through the cones in an S-shaped path, and then sprinted to the finish line. Two trials were performed, and completion time was recorded to the nearest 0.1 s.
Balance beam	Balance beam was assessed by timing the child as they walked from the start to the finish line with alternating steps and arms extended sideways. Two trials were performed, and completion time was recorded to the nearest 0.1 s.

Three identical bioelectrical impedance analyzers (InBody 570) were used for body composition measurements across all participants. No study-specific or diagnostic cutoff values were used to classify body fat mass or BFP. Segmental body composition data were retained as supplementary measurements.

Each participant in the intervention group will receive a weight management file, and children’s weight and height will be recorded at multiple time points throughout the intervention period. BMI will be calculated as weight (kg) divided by height squared (m²). According to the growth standards for Chinese children younger than 7 years (WS/T 423—2022), a healthy BMI for children is defined as greater than or equal to −2 SD but less than +1 SD; overweight is defined as BMI greater than or equal to +1 SD but less than +2 SD; and obesity is defined as BMI greater than or equal to +2 SD [36]. BMI-for-age *z* scores were calculated according to the 2006 World Health Organization Child Growth Standards for children aged ≤60 months and the 2007 World Health Organization Growth Reference for children aged ≥61 months, taking into account each child’s age and sex [37,38].

Blood pressure was evaluated using the Chinese sex-, age-, and height-specific reference standards recommended in the Chinese Guidelines for Hypertension Prevention and Treatment (2024 revision) [39]. Systolic and/or diastolic blood pressure at or above the 95th percentile was classified as elevated blood pressure at that assessment. Because a clinical diagnosis of childhood hypertension requires elevated measurements on 3 separate occasions, no diagnosis was made based on a single study visit. Blood pressure will also be analyzed as a continuous outcome to compare changes between groups over time.

All data will be collected by trained professionals using standardized equipment and procedures. Approximately 5% of students will be rechecked; if measurement errors exceed 10%, all students will be remeasured. Data will be double-entered and cleaned with EpiData 3.1 software (The EpiData Association) to ensure accuracy.

### Interview With Kindergarten Teachers

Trained professionals will conduct interviews with teachers from each class in the school to know the daily activity schedules of the participants within the kindergarten, including the activity times, types (climbing, ball games, role-playing, etc), intensity (low, medium, high), locations (indoors or outdoors), sitting duration, lunch type, meal time, sleep time, as well as personal activities (activity preferences), dining (food intake, dietary preferences, independence in eating), and sleep conditions (difficulty falling asleep, early waking, no napping, frequent waking during sleep, etc), in order to assess the physical activity, sleep conditions, and dietary situation of children in the kindergarten. The detailed content can be found in Multimedia Appendix 1.

Teacher interview responses concerning variables with predefined criteria (eg, whether the daily physical activity time in the kindergarten met the prespecified standard) will be coded as binary variables according to standardized coding rules.

### Parent Questionnaire

During the study period, each participating parent from the intervention group and the control group will be invited to complete a total of 2 electronic questionnaires at baseline and 12 months. The parent questionnaire 1 consists of a standardized scale module and a supplementary information module. The standardized scale module includes the Strengths and Difficulties Questionnaire [40], KIDMED Questionnaire [41], Children’s Sleep Habits Questionnaire [42], Child Feeding Questionnaire [43], and Diet Quality Questionnaire [44]. Each scale retains its original items intact and is scored and analyzed according to the original scoring method to assess children’s mental health, sleep habits, dietary behaviors, and dietary quality, as well as parents’ feeding behaviors. The supplementary information module is used to collect basic information such as the child’s gestational age at birth, delivery method, and physical condition; family characteristics such as the primary caregiver, parents’ educational level, and the mother’s pregnancy status; children’s and parents’ physical activities; children’s screen usage time; family meal habits; and parents’ awareness of the hazards of obesity and related dietary knowledge. The supplementary information module was analyzed separately and was not included in the scoring of the standardized scales. Parents’ physical activity was assessed using a self-reported questionnaire that recorded the duration of physical activity at different intensity levels, sedentary time, outdoor activity, and screen time during the specified recall period. Parents’ dietary intake was assessed using a food frequency questionnaire that primarily recorded the frequency of consumption of vegetables, fruits, sugar-sweetened beverages, fried foods, and snacks. Parents’ knowledge was assessed using a self-developed questionnaire that primarily evaluated their understanding of childhood obesity and healthy lifestyle behaviors. The knowledge score was expressed as the percentage of correctly answered items, with higher percentages indicating greater knowledge. All information was collected through questionnaires completed by the parents rather than through continuous or real-time monitoring.

For the follow-up at the primary school stage, we will redesign the questionnaire to suit the children’s circumstances at that time. The newly designed questionnaire will be named Parents Questionnaire 2.

### Ethical Considerations

The research program was endorsed by the Suzhou Center for Disease Control and Prevention Ethics Committee (SZCDC/IRB/PJ/2025‐07). This study adhered to ethical standards, obtaining informed consent from both participants and their parents. All methods were carried out in accordance with relevant guidelines and regulations.

To protect the privacy of participants and ensure the confidentiality of the data, all collected information was subjected to strict deidentification during the analysis process by replacing personal identification with unique alphanumeric codes.

No special provisions for compensation are made for this low-risk trial.

If, during the research process, any children are found to have abnormal blood pressure or other health problems that require further attention, the researchers will promptly inform their guardians and recommend that they seek evaluation and necessary treatment at a medical institution. The intervention is not expected to cause serious intervention-related adverse events, and no formal interim efficacy analysis or early stopping procedure is planned. Participant safety, recruitment, protocol adherence, withdrawals, and any adverse events will be monitored regularly by the principal investigator and the study team. Any serious or unexpected adverse event will be promptly reviewed and reported to the relevant ethics committee and institutional authorities in accordance with applicable requirements.

If there are any significant modifications to the research plan in the future for this study, a revised plan will be prepared. This revised plan will first be submitted to the ethics committee for approval. After approval, the research team will notify all researchers through a formal meeting and update the information simultaneously on the clinical trial registration center. If the modifications affect the rights and interests of the participants, the parents will be informed promptly and the informed consent form will be re-signed.

Any amendments to the study protocol will be submitted for approval prior to implementation. Written informed consent will be obtained from all children and their parents. All data collected will be entered into an electronic database with deidentified information. The database will be accessed only by designated staff with a password. The results will be disseminated through publication in peer-reviewed journals, presentation at conferences, and lay summaries provided to school staff and participants.

### Retrospective Registration

This trial was initiated with participant recruitment in March 2025 and completed retrospective registration in January 2026. The delay in registration was due to the research team’s insufficient understanding of the time requirements for trial registration. All study outcome indicators and corresponding assessment time points were predetermined in accordance with the research protocol before the trial registration, and the registration information was consistent with the research protocol. The research team did not add, delete, or modify outcome indicators based on the obtained data or preliminary research results.

### Sample Size Estimation

Sample size estimation was conducted using the formula: n_1_=n_2_=2 [(Zα+Zβ) σ/δ]². The significance level α was set at .05, and the confidence level (1 − β) was set at 80%. Based on similar previous literature, the overall SD of zBMI in the population was estimated at 1.4, with an anticipated difference in zBMI of −0.2 between the intervention and control groups [45]. Allowing for a 10% attrition rate, the minimum required sample size for each group was determined to be 674 students.

### Statistical Analyses

Statistical analysis will be conducted using SPSS software (version 25.0; IBM Corp) and R (version 4.3.3; R Foundation for Statistical Computing). All 2-sided *P* values less than .05 will be considered statistically significant.

Baseline characteristics will be described and compared for both the intervention and control groups at the participant level (eg, anthropometric indicators), school level (eg, school size), and family level (eg, parent questionnaires). For continuous data, the mean and standard deviation will be used when data are normally distributed, while the median and quantile will be reported for skewed distributions. Categorical variables will be summarized using frequencies and percentages.

All follow-up analyses will employ an intention-to-treat approach. Primary and secondary outcomes will be compared between the intervention and control groups at 6 months, 12 months, and subsequent follow-up periods using mixed-effects models appropriate to the type of outcome variable and the hierarchical structure of the data. The models will include intervention group, follow-up time, and the interaction between intervention group and follow-up time to assess differences in changes over time between the 2 groups.

For continuous outcomes, such as BMI, BMI *z* score, BFP, waist circumference, and chest circumference, linear mixed-effects models suitable for longitudinal repeated-measures data will be used. Adjusted mean differences between groups and their corresponding 95% CIs will be reported to assess the effectiveness of the intervention. For binary outcomes, such as overweight or obesity status and whether the amount of physical activity at school meets the recommended level, generalized linear mixed-effects models will be used. The proportions in each group at baseline and follow-up will be reported, together with adjusted odds ratios and their corresponding 95% CIs.

The adjusted analyses will take into account factors that may affect the study outcomes, including the child’s age and sex, the baseline value of the relevant outcome measure, family income, maternal education level, family structure, and kindergarten location. Random effects at the child and kindergarten levels will be included, where appropriate, to account for repeated measurements within the same child and correlations among children within the same kindergarten.

Given the multiple outcome measures and follow-up time points, appropriate adjustments will be applied to control for multiple comparisons. Primary and secondary outcomes will be distinguished when interpreting the results, and the potential impact of multiple comparisons will be carefully considered. The analysis results will primarily be presented as effect estimates and their 95% CIs, together with the corresponding *P* values.

The long-term annual follow-up data will be included in the longitudinal analyses together with the baseline, 6-month, and 12-month follow-up data to compare the trajectories of outcome changes between the intervention and control groups and to assess whether the intervention effects persist, weaken, or emerge with a delay over time. The same longitudinal mixed-effects modeling framework will be extended to the annual follow-up data, with the specific model selected according to the type and distribution of each outcome variable.

The completion status of each annual follow-up and the reasons for loss to follow-up will be reported. The primary analyses will be conducted using all available data under the intention-to-treat principle. If necessary, sensitivity analyses will be conducted to evaluate the potential effects of missing data and changes in relevant factors during the follow-up period on the long-term findings. The specific analysis methods will be prespecified in the formal statistical analysis plan.

### Outcomes

In Table 4, primary and key secondary outcomes are outlined, including data collection methods and timing of measurement. Although BMI cannot distinguish body fat mass from soft lean mass, it was selected as one of the primary outcomes because it is a standardized, widely used, and feasible measure for long-term follow-up and facilitates comparison across studies. BFP measured by bioelectrical impedance analysis and other anthropometric indicators were included to provide complementary information on adiposity and body shape.

**Table 4.** Primary outcomes and key secondary outcomes, data collection methods, and timing of measurement^a^.

		Timing of measurement						
Outcome level and measure	Data collection methods	Baseline	After 6 months	After 12 months	After 24 and 36 months	After 48 months	After 60 and 72 months	After 84 months
Primary outcome								
Healthy weight development								
Child BMI	Anthropometric data collected by research group	✓	✓	✓	✓	✓	✓	✓
Child BMI *z* score	Anthropometric data collected by research group	✓	✓	✓	✓	✓	✓	✓
Child body fat percentage	Bioimpedance data collected by research group	✓	✓	✓	✓	✓	✓	✓
Key secondary outcomes								
Healthy weight development								
Child overweight/obesity prevalence	Anthropometric data collected by research group	✓	✓	✓	✓	✓	✓	✓
Child chest circumference	Anthropometric data collected by research group	✓	✓	✓	✓	✓	✓	✓
Child waist circumference	Anthropometric data collected by research group	✓	✓	✓	✓	✓	✓	✓
Child hip circumference	Anthropometric data collected by research group	✓	✓	✓	✓	✓	✓	✓
Child body composition	Bioimpedance data collected by research group	✓	✓	✓	✓	✓	✓	✓
Child growth status	Anthropometric data collected by research group/measured by teachers or parents	✓	✓	✓	✓	✓	✓	✓
Child cardiovascular								
Child blood pressure	Anthropometric data collected by research group	✓	✓	✓	✓	✓	✓	✓
Child well-being								
Child mental health	Parent questionnaires^2^/Strengths and Difficulties Questionnaire [40]	✓	✓	✓		✓		✓
Child eating habits								
Child dietary intake	Parent questionnaires^2^/KIDMED Questionnaire [41]	✓	✓	✓		✓		✓
Child eating behavior	Parent questionnaires^2^/interview with teachers	✓	✓	✓		✓		✓
Child physical activity								
Child activity intensity	Parent questionnaires^2^/interview with teachers	✓	✓	✓		✓		✓
Child physical fitness	Anthropometric data collected by research group/interview with teachers	✓	✓	✓		✓		✓
Child screen use	Parent questionnaires^2^	✓	✓	✓		✓		✓
Child sleep patterns	Parent questionnaires^2^/Children’s Sleep Habits Questionnaire [42]	✓	✓	✓		✓		✓
Family food and meals								
Parental feeding practices	Parent questionnaires^2^/Child Feeding Questionnaire [43]	✓	✓	✓		✓		✓
Family meal habits	Parent questionnaires^2^/Diet Quality Questionnaire [44]	✓	✓	✓		✓		✓
Family physical activity								
Parental activity intensity	Parent questionnaires^1,2^	✓	✓	✓		✓		✓
Family screen use								
Parental screen	Parent questionnaires^1,2^	✓	✓	✓		✓		✓
Family healthy knowledge								
Parental healthy knowledge	Parent questionnaires^1,2^	✓	✓	✓		✓		✓

a Parent questionnaires1 will be used at baseline, after 6 months, and after 12 months. Parent questionnaires2 will be used between 24 and 84 months.

## Results

The baseline assessment was conducted from March to June 2025. All participants from the intervention and control schools will undergo these assessments. Follow-up assessments will be conducted once a year at 6 months, 12 months, and throughout the primary school years after the baseline assessment. All data will be managed by designated personnel, and the research data will be stored in locked filing cabinets in paper form.

The study received ethical approval in March 2025. This study sequentially implemented the intervention measures from March to June 2025. The intervention period for all participants was completed by June 2026. Following the intervention, participants will enter a noninterventional long-term follow-up period extending through June 2032, during which follow-up surveys will be conducted annually. Data collection and analysis are ongoing. Findings based on data collected during the intervention period are expected to be published in 2027, whereas analyses of the complete dataset, including the long-term follow-up data, are anticipated to be published in or after 2033.

## Discussion

### Principal Findings

In recent years, obesity among preschool children in China has risen significantly [46], particularly in economically developed urban areas where the problem is more pronounced. The increasing prevalence of childhood obesity is closely linked to urbanization, lifestyle changes, and shifts in dietary patterns [47]. In regions experiencing rapid economic growth, preschool children face a higher risk of overweight and obesity, largely due to higher energy intake and insufficient physical activity [48]. As a result, this issue has emerged as a major public health challenge that demands comprehensive interventions.

This study aims to develop a comprehensive intervention strategy to address overweight and obesity among preschool children in Suzhou. Grounded in the theories of SEM and SCT, the strategy integrates multilevel measures targeting individuals, families, schools, communities, and society as a whole. It further establishes a comprehensive prevention model involving the community, schools, families, and health care providers. The effectiveness and implementation of this approach will be evaluated, and if proven successful, it can be extended to other areas of Suzhou to promote healthy growth in children.

### Comparison With Prior Work

Although many intervention studies have addressed preschool obesity, most existing programs have notable limitations: they often lack long-term follow-up and fail to provide an integrated assessment of children’s development status and BFP. To date, no comprehensive intervention system has demonstrated both sustained effectiveness and thoroughness. Our research plan specifically targets preschool children and incorporates annual health tracking throughout the primary school years, aiming to build a robust, long-term weight management assessment system. This approach enables us to systematically observe the sustained effects of intervention measures on children’s growth and development, moving beyond simple weight monitoring to include precise BFP measurements. Notably, obesity and other common childhood health problems, such as myopia, often share risk factors and underlying mechanisms [49]. By recognizing these connections, our program offers a more holistic understanding of how interventions support overall child health, allowing for the prevention of multiple conditions and fostering well-rounded growth as a solid foundation for the future.

Our intervention measures emphasize both simplicity and effectiveness. We seamlessly integrate all strategies into children’s daily routines—diet, sleep, and exercise—making them easy to implement in both kindergarten and at home. These interventions naturally fit into the daily teaching process, allowing teachers to incorporate them without additional workload or overtime. This approach ensures sustainability and minimizes disruption.

### Limitations

As a research study, we should take into account some anticipated limitations. First, due to the long follow-up period of this study, there is inevitably a risk of loss to follow-up, which may lead to bias in the final analysis sample and affect the accuracy of the assessment of the long-term sustainability of the intervention measures. Second, although a comprehensive intervention approach was adopted, due to the limited sample size, the study may not be able to conduct in-depth analysis of specific subgroups (such as children with different birth weights or family economic backgrounds). Finally, since the dietary and exercise records of preschool children are mostly based on self-reports by parents or teachers, even with the use of validated measurement tools, there may still be recall bias or social desirability bias, which may have a subtle impact on the objectivity of the research results.

### Conclusions

In summary, this research has the potential to make a significant impact on the prevention and control of childhood obesity in Suzhou. The findings will help advance intervention research and offer a practical, replicable program for addressing childhood obesity. In the long term, this study is also expected to contribute valuable insights to the broader development of intervention research for childhood obesity in China.

## Supplementary material

10.2196/94697 Multimedia Appendix 1 Parent questionnaire.

10.2196/94697 Checklist 1 SPIRIT 2025 checklist.

## Abbreviations

BFP: body fat percentage
SCT: Social Cognitive Theory
SEM: Social Ecological Model
