# Supplementary material for: A Multifaceted Intervention to Reduce Obesity Among Preschool Children in Suzhou City: Protocol for a Randomized Controlled Trial
Source: JMIR Res Protoc. 2026 Oct 21;15:e94697. doi: 10.2196/94697 (PMC13686611; doi:10.2196/94697)
Supplement: Supplementary file 1 [file resprot-v15-e94697-s001.docx]

**Weight management questionnaire for preschool children in Suzhou (teacher interview version)**

|  | During the last week, the child was in kindergarten and averaged daily physical activity | Time |
| --- | --- | --- |
| 1 | Low-intensity physical activity time, such as walking slowly on a flat surface, light physical activity while standing, such as standing with toys, making the bed, playing an instrument, etc | __ hours __ minutes |
| 2 | Moderate intensity physical activity time, such as brisk walking, throwing and picking up the ball, jogging, cycling at normal speed, skating, limp gymnastics, gymnastics, dancing, stair climbing, scooter, etc  (The child sweats slightly, breathes more quickly, can only speak short sentences during exercise, can not fully express long sentences) | __ hours __ minutes |
| 3 | High intensity physical activity time, such as fast running, intense ball playing, intense kicking, fast riding, fast rope skipping, etc  (The child is sweating, breathing fast, labored and unable to talk.) | __ hours __ minutes |
| 4 | Time spent outdoors during the day | __ hours __ minutes |
| 5 | Sedentary time indoors, such as sitting watching TV, colouring, doing puzzles, reading or listening to others reading, and playing indoors in a fixed manner, such as playing board games | __ hours __ minutes |
| 6 | Nap in the afternoon | __ hours __ minutes |

|  | **During the last week, children in kindergarten, average daily physical activity preferences as well as dietary preferences** | **Choice** |
| --- | --- | --- |
| 7. | Preference for activities | 1. Be static 2. Partial dynamics 3. Not obvious |
| 8. | Food intake assessment | 1. Very few 2. Less 3. normal 4. more |
| 9. | Dietary preferences | 1.More meat  2.More hormones  3.More staple food |
